# Understanding the Ethnobotany, Chemistry, Pharmacology, and Distribution of Genus *Hydnora* (Aristolochiaceae)

**DOI:** 10.3390/plants10030494

**Published:** 2021-03-05

**Authors:** Elijah Mbandi Mkala, Moses Mutuse Mutungi, Elizabeth Syowai Mutinda, Millicent Akinyi Oulo, Vincent Okelo Wanga, Geoffrey Mwachala, Guang-Wan Hu

**Affiliations:** 1 CAS Key Laboratory of Plant Germplasm Enhancement and Specialty Agriculture, Wuhan Botanical Garden, Chinese Academy of Sciences, Wuhan 430074, China; mkala@wbgcas.cn (E.M.M.); mutungi.moses7@gmail.com (M.M.M.); elizabeth@wbgcas.cn (E.S.M.); millicentoulo@gmail.com (M.A.O.); vincentokelo@gmail.com (V.O.W.); 2 Sino-Africa Joint Research Center, Chinese Academy of Sciences, Wuhan 430074, China; 3 University of Chinese Academy of Sciences, Beijing 100049, China; 4 East African Herbarium, National Museums of Kenya, P.O. Box 451660-0100 Nairobi, Kenya; gmwachala@museums.or.ke

**Keywords:** *Hydnora*, geographical distribution, ethnopharmacology, chemistry, biological activities

## Abstract

The genus *Hydnora* (Hydnoraceae) is one of the basal angiosperms in the order Piperales, found in the semi-arid regions of Africa, and the Southern Arabian Peninsula. Plants in this genus play essential roles in communities around the world as revealed by various studies. Currently, there are eight species of the genus *Hydnora*; seven in Africa and one in the Arabian Peninsula. Notably, *Hydnora abyssinica* A.Br. and *Hydnora africana* Thunb. are widely distributed compared to other species. They are widely used for their medicinal and nutritional values. The information on ethnobotany, chemistry, pharmacology, and distribution of genus *Hydnora* was gathered using phytochemical and ethnobotanical books, electronic sources, and published articles. Preliminary phytochemical screening shows that flavonoids, phenolics, proanthocyanidins, and tannins are the main compounds in *H. abyssinica* and *H. africana*. Furthermore, 11 compounds have been isolated from *H. abyssinica*. The biological activities of *H. abyssinica* and *H. africana* have been reported. They include antibacterial, antiproliferative, antioxidant, antidiarrhea, and antifungal potentials. Despite the *Hydnora* species being practiced in ancient folkloric medicine, their traditional uses and pharmacological value are poorly documented. Based on the available information on ethnobotany, phytochemistry, pharmacology, and distribution, we aim to provide research gaps and challenges for a better understanding of this genus. This may be resourceful in the development of effective phytomedicines, and aid in conservation. The available studies on this genus on some aspects such as phytochemistry, pharmacological activities, and distribution are under-reported hence the need for further research.

## 1. Introduction

Hydnoraceae is grouped under the Aristolochiaceae family and consists of parasitic plants characterized by large flowers and lacking leaves [1,2]. They are native and distributed in arid and semi-arid parts of Africa and Asia. This family contains two genera; *Hydnora* and *Prosopanche*, named among the oldest parasitic lineages [1,2,3]. *Prosopanche* is naturally found in Central America and South America, whereas *Hydnora* is indigenous to Africa, Madagascar, and the Arabian Peninsula [2,3,4].

*Hydnora* consists of eight species, namely, *H. visseri* Bolin, E. Maass, and Musselman [4,5], *H. arabica* [1], *H. abyssinica* A.Br. [6,7], *H. esculenta* Jum. and H. Perrier [4,7], *H. africana* Thunb. [8], *H. triceps* Drège and E. Mey [9], *H. sinandevu* Beentje and Q. Luke [10,11], and *H. longicollis* (Welw.) Bolin [5,12]. Recently, two new *Hydnora* species have been identified in different locations [1,5].

Since the first printed note of *Hydnora* and its use by Thunberg and Ludwig Pappe in 1847 [13,14,15], its species have been used in traditional medicine and as a source of food and tannins in local communities of Africa and the Arabian Peninsula. For example, the roots of *H*. *abyssinica* and *H. africana* were introduced and commercialized as a traditional medicine in South Africa and Southern Mozambique [14,16,17,18]. In Uganda, Sudan, and Kenya, some *Hydnora* species are used as a source of food and for the treatment of various diseases [16,19,20]. *H. abyssinica* decoction is used as a remedy for inflammation, tonsillitis, and dysentery in Sudan [20,21]. In Oman, the fruits of *H. abyssinica* are used as food and in tanning leather [22]. In Eastern Ethiopia, this plant (*H. abyssinica*) is used to treat diarrhea, hemorrhage, wounds, and mouth infections [23]. Moreover, the flowers of *H. abyssinica* are used as wild food and in traditional medicine in South Yemen [24]. Therapeutic properties, for instance, antibacterial, antifungal, antioxidant, and antiproliferative activities, have been reported in *H. abyssinica* and *H. africana* [25,26,27,28]. Regardless of the ethnobotanical value and medicinal significance of the *Hydnora* species, little effort has been made to integrate all of the relevant available data.

Despite some *Hydnora* species being used in traditional medicine, not much information is available on the ethnobotany of other species since most of them have been hardly studied. Moreover, the distribution of *Hydnora* species in Africa and other parts of the world remains unclear. These challenges are perhaps propelled by a scanty distribution of *Hydnora* species, insufficient sample collection, and poor preservation methods [6,11,12]. Due to their mushroom-like appearance, they can be confused with each other, posing a taxonomic challenge. Thus, a worldwide approach and comprehensive multidisciplinary research are essential to untangle the systematics, distribution, uses, and applications of this underexplored genus. Only a few scientific reports exist for *H. abyssinica* and *H. africana*; hence, further research is required, especially regarding the other hardly studied species in this genus. In this current study, we provided more insights and in-depth investigations on traditional and pharmacological uses of this genus, as well as its distribution, which will help in conservation as an alternative therapeutic and food source. Therefore, this review aimed to summarize up-to-date ethnobotanical uses, chemistry, pharmacology, and distribution of the *Hydnora* species, and also point out possible research gaps.

## 2. Materials and Methods

**Figure 1:**
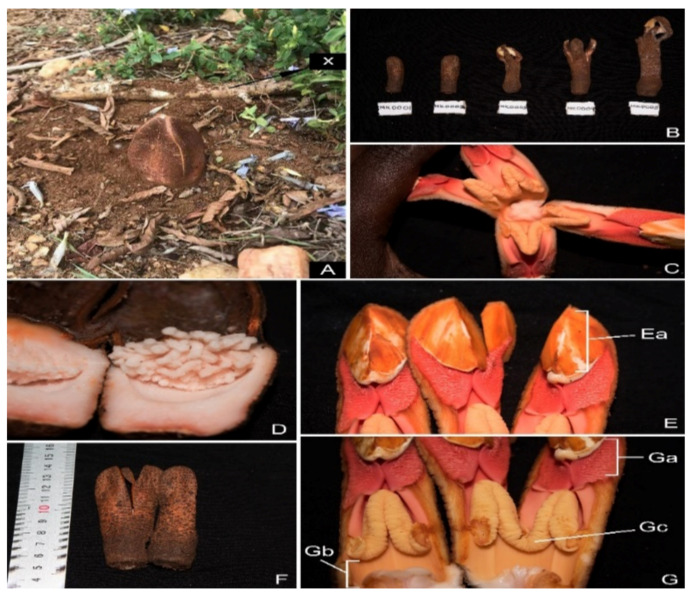
Flower of *Hydnora abyssinica*: (**A**) *H. abyssinica* plant growing, (**B**) images of other young *H. abyssinica* plant, (**C**) internal part of the flower, (**D**) fruit containing seeds white like rice, (**E**–**G**) floral parts; **Ea**—osmophores, **Ga**—androecial part, **Gc**—antheral rings, (**F**) outer part of the *H. abyssinica* plant.

This review employed literature published before December 2020 on the ethnobotanical uses, chemistry, and pharmacological activities of bioactive compounds from the genus *Hydnora*. To understand the worldwide distribution and other information on the *Hydnora* species, data were obtained from the following online data sources from February 2020: Regional floras (flora of Tropical East Africa) [11,29,30], world flora online [31], original species descriptions [1,5,7,9], and virtual online databases, e.g., African Plant Database (APD), Global Biodiversity Information Facility (GBIF) [32], International Plant Names Index (IPNI) [33], and Plants of the world online [34]. For some species, circumscriptions were assessed by reviewing the types of specimens on JSTOR Global Plants [35], Kew Herbarium Catalogue [36], World Checklist of Selected Plant Families [37], The Plant List [38], and a community for naturalists [39]. The *H. abyssinica* specimen was collected from Mt. Kasigau, Taita Taveta County, Kenya (SAJIT-Mkala 0001, −3.1941° N, 38.4997° E (Figure 1). The voucher specimens were deposited at Wuhan Botanical Garden Herbarium, China, and other herbarium collections (e.g., B, K, W, EA, BM, K, NBG, PREUS, GBG, FT, LISU, P, and BM), as part of ongoing research. An extensive search for ethnobotanical uses, pharmacology, and chemistry of *Hydnora* was made using published articles, journal magazines, Ph.D. and MSc dissertations, conference papers, available data from herbaria, and books published in English. Several papers were obtained from published research articles of the genus *Hydnora*. Web of Science, Google Scholar, Science Direct, PubMed, and SciFinder databases further facilitated our study using keywords, for example; *Hydnora,* pharmacological activities, phytochemistry, and ethnobotanical, without a specific time limit. Species details were named as per collector, species number, and the herbarium. The plant list and Plants of the world were used in the verification of species names. For the classification of families, we used the Angiosperm phylogeny group classification system (AGP IV) [40]. Chemical structures were drawn using ChemBio Draw Ultra version 14.0. Excel @ 2016 Microsoft Corporation and Adobe Illustrator version (2020-24.1) was used to draw all figures and graphs.

Published articles, reviews, and dissertations were searched online using titles and keywords, obtaining a total of sixty-five for *Hydnora*. Thirty-one papers were retrieved and used in pharmacological activities. The total number of records identified from all citation sources was 172 and 92 after identifying duplicated data. Google Scholar had the highest number of citations with eighty-four, followed by the web of science with twelve.

## 3. Botanical Features and Taxonomy

*Hydnora* species are underground holoparasitic herbs and their rhizomes are attached to the host. The rhizomes are 1 cm wide, 4–5 angled, terete, and sometimes flattened. The periderm is well developed, brick-red, apart from the tip of the rhizome. Fresh rhizomes are pink to flesh-red with gummy exudate, which is very bitter and severe. The whole rhizome is enclosed with warty outgrowths of haustoria which can be spread sometimes regularly up to 0.8 mm long or unevenly, and less than 0.5 mm long, except at the tip. The rhizomes are covered with latent and active outgrowths in groups of 2–4. Flowers are 3, 4, or 5 merous. The floral envelope is clear and rests on the ground; sometimes they are not reflexed, and they open flowers by separating the floral envelope. Flowers vary in size from 5 to 25 cm depending on the distance of the rhizome from the ground and pedicel (4–9 cm). The ovary is inferior, unilocular, and highly enfolded pendant placentae. The stigma is sessile with distinctive indentations on the surface. Stamens are attached at the base, and anthers are 2.5–3 × 2–2.5 cm in length. Pollen adhering to anthers is very sticky. The floral envelope is 6–8 cm; some species have “bait bodies” in between the internal margin of the envelopes (lobes) while some contain well-developed petals that are concave in shape. The fruits are fleshy, globose, 10–15 cm wide, with many seeds. The mature pedicel is very short and easily disconnected from the rhizome. The outer skin is scaly, and the internal layer is white. The placenta is similar to the internal layer of the pericarp in texture. Seeds are brown, very hard, irregularly shaped, and oblong to globose [1,4,5]. Their pollination mechanism generally follows the same floral phenology as that of *H. africana* and *H. esculenta* [7,8].

Data from literature, world herbarium, and online tools were used to confirm the accepted names and synonyms [1,12,34,38]. The species were confirmed as follows; *H. africana* Thunb. (= *H. africana* var. *longicollis* Welw.), *H. esculenta* Jum. and H. Perrier, *H. abyssinica* A. Braun (= *H. johannis* Becc. = *H. solmsiana* Dinter), and *H. triceps* Drège and Meyer, as distinct species. Afterward, *H. sinandevu* Beentje and Q. Luke was designated to a collection from Kenya and Tanzania [11,30].

The taxonomic placement of the Hydnoraceae family is unresolved and studies have classified it differently using a molecular approach. Regardless of them lacking some morphological features (leaves), the Hydnoraceae family was grouped within Rafflesiales [41]. Another study compared Hydnorales and Rafflesiales to the Aristolochiaceae family [42]. Based on floral characteristics, geographic distributions, and embryology, Hydnoraceae was connected to the Annonaceae and Mistrastemonaceae families [43]. Using the molecular approach, the Hydnoraceae family was grouped in Piperales under the Aristolochiaceae family but did not include Asaroideae [2]. The relationship of families in order Piperales is unresolved in the Angiosperm phylogeny group classification (APG III) system [44]. Some research studies support the placement of Hydnoraceae within the Aristolochiaceae family [45,46]. This has contributed to the placement of Hydnoraceae within Aristolochiaceae due to their past paraphyly APG IV [40]. However, a recent study grouped the Hydnoraceae family as a sister group to the Winteraceae family (*Drimys granadensis*) because of the long branching problem [45]. Another study indicated that the Hydnoraceae family formed a monophyletic clade with a low bootstrap support value of 13%, which cannot justify the grouping of Hydnoraceae [3]. To date, only *H. visseri* and *H. abyssinica* complete genomes have been studied in this genus (5, Mkala et al. in press). In genus *Prosopanche*, only the *Prosopanche americana* genome has been documented [3]. More studies on the genomes of the remaining species need to be analyzed. This will enable researchers to match particular characteristics observed in plants to their underlying genetic features.

## 4. Habitat and Ecology of *Hydnora* Species

Most of the *Hydnora* species are found in the semi-arid and desert regions in Africa and the Arabian Peninsula [1,2,3,4,5,6]. They are obligate parasites on various host plant species belonging to the Fabaceae and Euphorbiaceae families (Table 1).

## 5. Host Specificity Concerning Species Distribution

**Figure 2:**
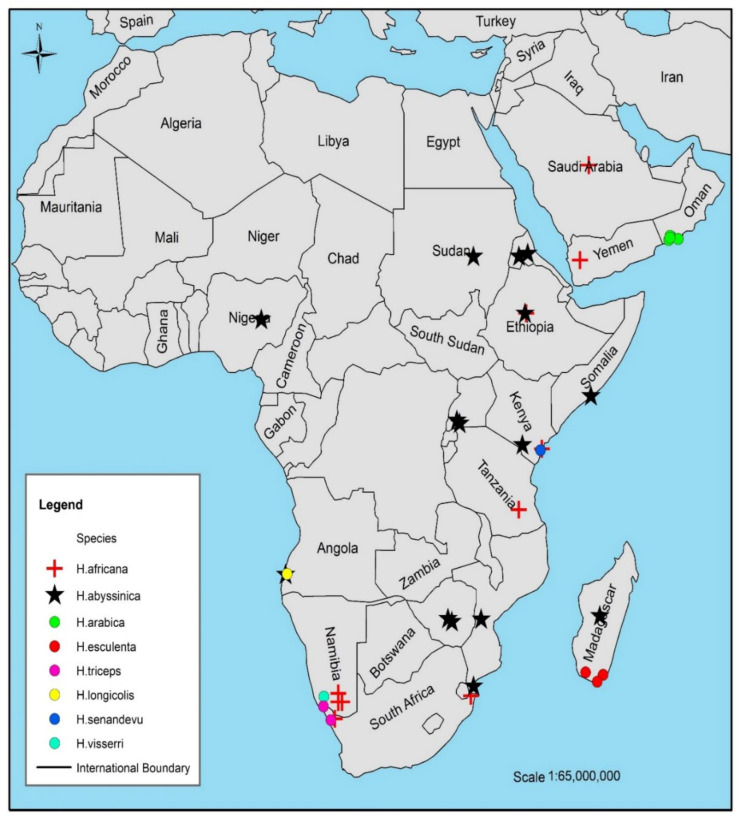
Distribution of *Hydnora* species in Africa and Arabian Peninsula adapted from [50].

*Hydnora* species have been reported in South Africa, Madagascar, Uganda, Mozambique, Swaziland, Botswana, Kenya, Sudan, Ethiopia, Tanzania, Angola, Somalia, Namibia, and the Arabian Peninsula (Figure 2) [1,6,7,8,9,11,24,28,40,43,44]. Host-based speciation appears to be a dynamic aspect in the evolution of *Hydnora* and this is reinforced by vicariance and changes in phenology. The wide distribution range of the Euphorbiaceae and Fabaceae families may also have led to the subsequent increase in the distribution range of the *Hydnora* species. These species are host-specific (Table 1). *H. africana*, *H. longicollis*, and *H. visseri* showed high host-specificity with *Euphorbia* species. *H. abyssinica, H. arabica,* and *H. esculenta* are host-specific to Fabaceae, while *H. triceps* grow on both Euphorbiaceae and Zygophyllaceae families. The variation of host dependency of these species to their host plants has not been revealed whether they exchange genes that help them to perform other metabolic activities, or they entirely depend on their hosts for nutrients. The distribution range of this genus is increasing due to their apparent host. *H. abyssinica* and *H. africana* have shown a higher distribution range compared to the other species (Figure 2). Furthermore, *H. longicollis* is not well-known due to its inaccessibility [5]. Recently, this species was collected from Namibia growing on *Euphorbia*; however, very little information on this species is available in the Kew herbarium (http://specimens.kew.org/herbarium/29047.275 (20 December 2020) [47,48]. *H. esculenta* is only endemic in Madagascar and mostly found in association with the invasive tree, *Pithecellobium dulce*. The distribution range of *H. esculenta* is increasing due to the spread of *P. dulce* in riparian areas and disturbed habitats in Southern Madagascar [4]. *H. triceps* is only endemic in Northwestern Cape and Southern Namibia because of the restricted distribution of the host. It depends completely on *Euphorbia dregeana*. A few samples have been collected since its discovery, and so it remains poorly studied [9]. *H. visseri* is distributed from Namibia to the Northern Cape Province in South Africa. Its distribution is restricted around the Orange River, where its host plants include *Euphorbia gummifera* Boiss. and *E. gregaria* Marloth [5]. *H. sinandevu* is native in Kenya and Tanzania [11]. *H. arabica* is distributed from Southern Oman (Dhofar region) to Yemen on Acacia species [1]. *H. africana* is distributed in Namibia, Swaziland, Nigeria, Kenya, Zimbabwe, Uganda, Madagascar, Saudi Arabia, South Africa, and Ethiopia [8,12,25,43,47,48]. *H. abyssinica* is the most widely distributed species in Namibia, Northern Botswana, Zimbabwe, Zaire, Tanzania, Kenya, Ethiopia, Somalia, Sudan, and the Arabian Peninsula [11,23,24,29,40,49,50,51].

### 5.1. Finding the Hydnora Species in the Wild

*Hydnora* species are rarely found and collected because of their uneven distribution and seasonal flowering. Based on the existing documentation, they are scarce and poorly preserved posing a taxonomic challenge [49]. *Hydnora* features that are mainly used by gatherers to identify *Hydnora* species include rhizome (protrude after rain season), and presence of flowers. Most gatherers prefer collecting the *Hydnora* species in areas they collected them before.

### 5.2. Discovery of New Sites of Hydnora Species in Kenya

*H. abyssinica* was recently collected in Kenya at Mt. Kasigau. Its local name is “tuka”, and the local community uses it as a source of food (Figure 1). In Kenya, *Hydnora* species are found in Kakamega, Mbololo, Mwakitau, Kishushe, Sisera, Ronge, Chyulu Hills, Arabuko Sokoke, Galole (Tana River), Mau, Baringo South, Maungu (Tsavo), and Narok (Rift Valley).

## 6. Approaches to Conservation Status

Based on the literature, the conservation status of *H. arabica* in Southern Oman is stated as less threatened; however, it is rare in Yemen and Saudi Arabia [1]. *H. abyssinica* in South Africa is stated as least threatened, as well as in East Africa Flora [6]. There is a lack of information on *H. sinandevu* conservation status [29]. *H. visseri* and *H. africana* have also been documented as less threatened in the South African online red list database [51]. No documentation is available for *H. longicollis*, while *H. esculenta* is stated endemic in Madagascar but there is no information related to its conservation. Similarly, there is no information available on the conservation status of *H. triceps*. The risk assessment of the Hydnoraceae family is not known in the International Union for Conservation of Nature (IUCN) and the number of *Hydnora* species in Herbaria is limited [52,53,54]. There is a need for more efforts to establish a proper habitat and area coverage which has been ignored so far. Effective conservation of *Hydnora* species relies on the existing knowledge of the genetic structure of natural populations and their habitat protection. Their possibility of being propagated as indicated on *H. africana* deserves more research [51].

## 7. Ethnobotany

Few studies have been done and documented on the ethnobotanical value of the *Hydnora* species. *H. triceps*, *H. sinandevu*, *H. visseri*, *H. esculenta*, and *H. longicollis* are poorly documented with little information known about them because of their subterranean behavior, and some grow in unsafe zones (*H. longicollis* in Angola). They are characterized by a unique flowering system and since they are sparsely distributed, it requires a lot of work to be done to identify more of these species. Some of the existing studies indicate that the roots were used in traditional medicine for alleviating diarrhea, possibly due to their high concentration of tannins.

### 7.1. Species Uses

*Hydnora* plants are a source of food for wild animals, particularly fruits [55]. According to the herbarium label (Bally 7694 (K), rhinoceroses were reported to feed on *H. abyssinica*. Extensive diggings of *H. abyssinica* by elephants have also been observed in Etosha Pan National Park [56]. In the Namib Desert, *H. africana* roots are fed on by wild animals because of their high water content [4,57].

The fruits of *H. abyssinica* are also edible by humans. Dried roots are used for charcoal preparation and tanning leather in Sudan [19,58]. In Kenya and Uganda, this species is used for food by the Pakwacha and Pokot communities [55]. The reddish-brown subterranean fruit, which resembles fresh lean meat is the edible part. Moreover, it is used to treat various diseases when combined with other medicinal plants such as paralysis, diabetes, hiccups, fever, insomnia, hypertension, measles, hemorrhoids, and diarrhea [54]. In Tanzania, it is used as a remedy for throat inflammations and swollen tonsils, whereas in Angola it is used as a styptic remedy [59]. The flowers were traditionally used as food and to treat gastrointestinal diseases and cancer in Yemen [24].

*H. africana* has been utilized for food, leather tanning, and fishing nets preservation [14]. Alternatively, *H. africana* decoction is used to treat dysentery, chronic diarrhea, persistent stomach cramps, and as a coagulant [51]. Infusions have been used as a face cleanser to treat acne [14,51]. Additionally, the root extracts have been used for alleviating inflammation of the throat amongst local communities in South Africa [4].

Both *H. abyssinica* and *H. africana* species have been used as antidiarrhea agents because of their high tannin quantities [4,14]. *H. arabica* is used as food by Jibbali settlers in Oman [1]. *H. visseri* fruits are edible [5]. *H. esculenta* was traditionally used as food, and for tanning leather [4,60]. Similarly, since it is rich in tannins, it has also been used as an astringent traditional medicine to treat diarrhea. *H. sinandevu* is used for the treatment of throat infections [51]. Generally, *Hydnora* species have been commercialized and they are readily available in the market [8,9,10,11,12]. Similarly, *H. triceps* and *H. longicollis* are only used as a source of food [5]. The uses of *Hydnora* species are summarized in Table 2.

### 7.2. Summary of Uses

**Figure 3:**
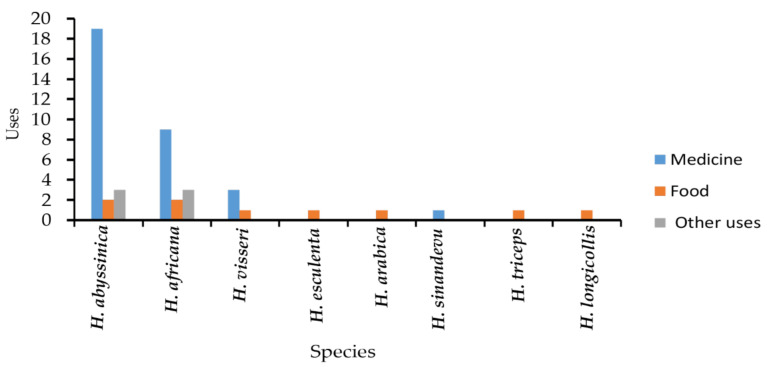
Ethnobotanical and other uses of *Hydnora* species in Africa and the Arabian Peninsula.

**Figure 4:**
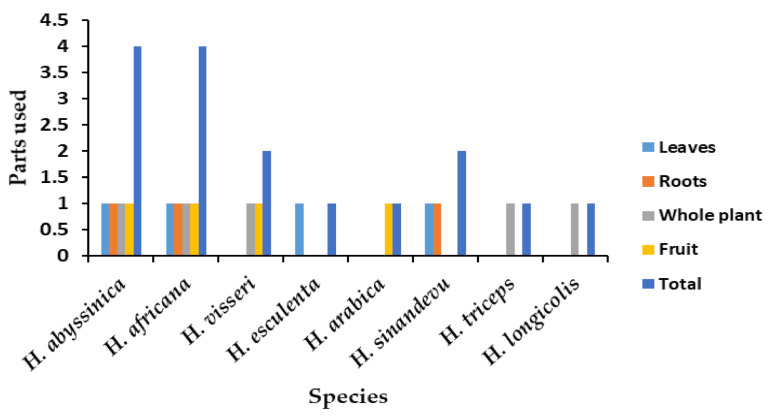
Utilized plant parts of *Hydnora* species.

A total of seven *Hydnora* species (87% of the genus) were found to have ethnobotanical and other usages (charcoal making and fishnet preservation). All species are limited to Africa (seven species of the total eight) and the Arabian Peninsula (two species of the total eight species). Their uses were divided into the following categories (with the corresponding number of species and percentages in brackets), medicinal (four species; 67.39%), food (eight species; 19.56%), and other usages (two species: 13.04%), as shown in Figure 3. The most utilized parts for medicinal purposes are indicated (Figure 4).

#### Most Used Species

*H. abyssinica* is widely used for medicinal purposes and food. It has 24 uses followed by *H. africana*, which has 11 uses (Figure 3).

### 7.3. Traditional Medicine

**Figure 5:**
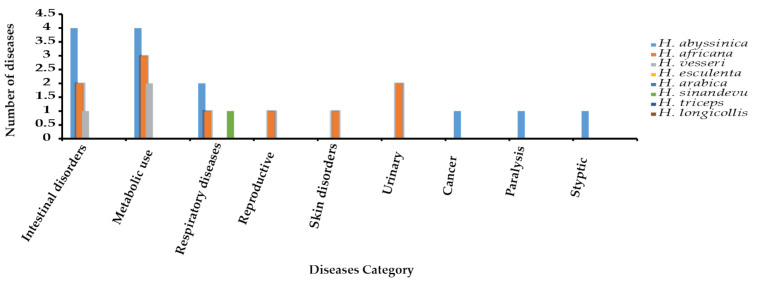
Diseases treated using *Hydnora* species in Africa and the Arabian Peninsula.

Four species were used, both separately and as part of the mixture to treat 20 different diseases. *H. abyssinica* dominated the literature with 19 medicinal uses, followed by *H. africana* (nine uses), (Figure 3). Diseases are categorized according to the body parts affected. The plants are prepared and used as decoctions, infusions, and powders to treat intestinal, metabolic, respiratory disorders, reproductive, skin, urinary, cancer, paralysis, and styptic diseases (Figure 5).

## 8. Chemical Composition and Biological Activities

*Hydnora* species serve as food for both humans and animals. Local communities use the roots, rhizomes, and whole plants for medicine preparations [58]. Despite their use in folkloric medicine, their chemical profiles and biological significance are not yet well established. Generally, there is limited documented evidence on this genus as some of its species are hardly studied.

### 8.1. Extraction Methods Used in Hydnora Species

Selecting an appropriate method for extraction depends on the bioactive compounds being targeted [61]. To enhance the extraction process, plant samples should first be pulverized to increase the surface area of sample contact with the solvent method. Moreover, solvents of varied polarities are used in the extraction process. Some of these solvents include chloroform, ethanol, water, methanol, dichloromethane, ethyl acetate, and n-butanol. For example, a previous study conducted by Koffi et al. [62] discovered that ethanol was more effective in the extraction of many phenolic compounds from walnut fruits as compared to methanol. Compounds tend to dissolve in these solvents depending on their polarity [61]. Currently, commonly used extraction methods include ultrasound-assisted, Soxhlet, percolation, and pressurized solvent. In this regard, the main extraction procedures used for the extraction of *Hydnora* plant samples are soaking, Soxhlet, ultrasound-assisted extraction, and maceration [28,63,64].

### 8.2. Phytochemistry

Understanding the phytochemical composition and content of *Hydnora* species is necessary to substantiate their ethnopharmacological uses. Moreover, characterizing their chemical components would facilitate the proper dispensing of their crude preparations. Currently, only a few studies are available on the pharmacological significance of the *Hydnora* species and their phytochemical composition. *H. abyssinica* is the only species that has been widely explored in this genus.

Different experimental techniques have been conducted to determine the phytochemical profile of these species, e.g., Qualitative chemical tests (colorimetrically), preliminary phytochemical screening, X-ray fluorescence (XRF), thin-layer chromatography (TLC), Fourier-transform infrared spectroscopy (FTIR), gas chromatography-mass spectrometry (GC-MS), and nuclear magnetic resonance (NMR). Further, quantities of total tannins, flavonoids, and phenolics have been evaluated. Preliminary chemical screening tests confirmed the presence of diterpenes, alkaloids, fatty acids, glycosides, triterpenes, flavonoids, saponins, sugars, steroids, anthraquinones, and polyphenols in *H. abyssinica* [65,66,67,68]. Similarly, flavonoids, alkaloids, saponins, steroids, tannins, and terpenoids were reported in *H. africana* [26,27,69]. Indeed, FTIR analysis results confirmed the presence of phenolics in *H. abyssinica* roots, whereas XRF analysis confirmed traces of mineral elements such as Na, Al, Mg, Si. P, Fe, Cu, and Zn [21]. Additionally, the low ash content of 3.81% signified low inorganic composition in this sample.

**Figure 6:**
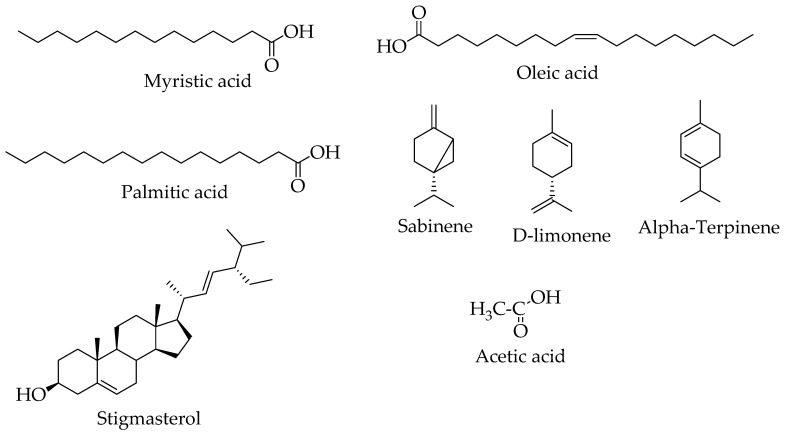
Chemical structures of *H. abyssinica* characterized using gas chromatography coupled with mass spectrometry (GC-MS) analysis.

**Figure 7:**
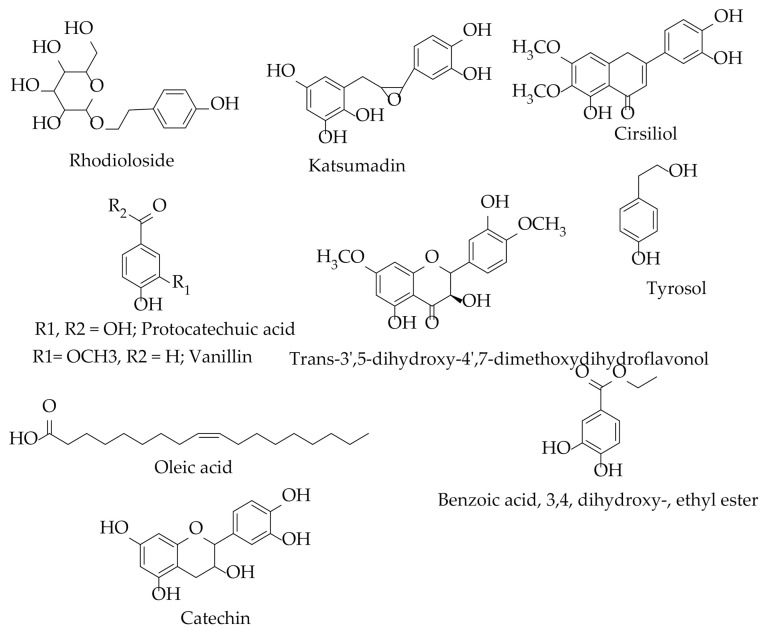
Chemical structures of isolated compounds from *H. abyssinica.*

Gas chromatography coupled with mass spectrometry (GC-MS) is an ideal analytical technique reported to analyze less polar and volatile compounds [70]. Only a few compounds of pharmacological importance have been characterized in *H. abyssinica* using this technique. They include myristic acid, oleic acid, palmitic acid, sabinene, acetic acid, γ-Terpinene, D-limonene, stigmasterol, and α-Terpinene [20,24,71] (Table 3, Figure 6). Furthermore, 11 compounds have been characterized and their structures elucidated using NMR (Table 3, Figure 7). They include katsumadin, rhodioloside, catechin, tyrosol, cirsiliol, oleic acid, trans 3′,5-dihydroxy-4′,7-dimethoxyflavonol, benzoic acid, 3, 4, dihydroxy-, ethyl ester, 2-hydroxyhexadecyl ester, vanillin, and protocatechuic acid. Additionally, it is worth noting that there has been no liquid chromatography-mass spectrometry work performed on all *Hydnora* species. Similarly, no phytochemical studies have been conducted on the other six *Hydnora* species, which is an opportunity for further exploration.

Crude drugs consist of multiple chemical species but only traces of these species contribute to their beneficial or harmful effects as well as potency. The content/yield of compounds in medicinal herbs and plants is believed to influence their biological effects [72]. Thus, estimation of compounds yield in these species is important. In this regard, total flavonoids, tannins, phenolics, anthocyanidins, saponins, and alkaloids contents have been estimated and reported in some *Hydnora* species. Elhassan et al. [64] reported high TFC, TTC, and TPC of 1163.43 mg QE/mg, 276.6 mg TAE/mg extract, and 5061.13 mg GAE/mg, respectively, in rhizome ethanol extract of *H. abyssinica*. A high TPC yield of 662.10 ± 14.4 and 661.58 ± 43.9 mg tannin acid/g was reported in water and ethanol extracts of *H. abyssinica* roots, respectively [73]. A recent study reported low yields of flavonoids, polyphenolics, and tannins in methanol, methanol-dichloromethane, and water fractions of *H. abyssinica* rhizomes [28]. A similar study was conducted on *H. abyssinica* roots which reported that total phenolics were the most abundant compounds ranging from 14.40 to 15.50 mg/g while alkaloids were the least abundant, ranging from 0.19 to 0.26 mg/g [26]. The slight variation in the yield contents may be linked to the difference in geographical areas where the plant samples were collected, and different extraction methods used.

### 8.3. Pharmacology of Hydnora Species

Both modern and traditional formulations complement each other in that they have a specific active component/s that take part in physiological effect once ingested or in bioassays. The presence of phytocompounds in crude preparation modulates biological activities by interacting synergistically with other test components, thus lowering any adverse reactions. Antiproliferative, antioxidant, anti-fungal, antidiarrhea, and antibacterial potentials have been reported in *H. abyssinica* and *H. africana*. Folkloric medicinal practices are currently being recognized in the search of new drugs as most natural products and other botanicals are regarded safe with minimal adverse effects if any [75].

In modern medicine, biological assays have been conducted to evaluate the antioxidant, antifungal, antiproliferative, antidiarrhea, and antibacterial potentials of these species.

### 8.4. Antibacterial and Antifungal Activities

*Hydnora* species are ascribed with good antibacterial and antifungal activities as shown by various experiments. Particularly, *H. abyssinica* has been extensively studied for antibacterial properties. Solvents of different polarities (water, methanol, dichloromethane, and ethanol) have been used to determine fungal and bacterial growth inhibition. Micro dilution, cup-plate diffusion, and agar disc diffusion were the most used antibacterial techniques. Both Gram-positive and Gram-negative are commonly used in most experimental tests. Some of the frequent bacteria strains used include *Escherichia coli*, *Staphylococcus aureus*, *Aspergillus aureus,* and *Bacillus subtilis*. Additionally, activity results were given in minimum inhibitory concentration (MIC). In plants, antibacterial activity is low if the minimum inhibitory concentration is greater than 625 µg/mL, high when the MIC is less than 100 µg/mL, and moderate if the MIC is between 100 µg/mL and 625 µg/mL [76]. This criterion is normally applied in deducing both antibacterial and antifungal activities. The antifungal and antibacterial activities of *Hydnora* species are reported in Table 4.

### 8.5. Antioxidant Activity

Pro-oxidants such as reactive oxygen species (ROS) are generated from pollutants from environs and also intracellularly due to stress and physiological activities in mitochondria [77]. Polyphenolics ubiquitously generated in plants are regarded as natural antioxidants. Antioxidants react with the free radicals, suppressing their deleterious effects and thus establishing cell homeostasis. Some of the mechanisms by which extracts from plants exhibit antioxidant properties include peroxidation of lipids, hydroxyl, oxygen radical scavenging, and metal chelation [78]. 2, 2-diphenyl-1-picrylhydrazyl (DPPH) and 2, 2′-azino-bis 3-ethylbenzthiazoline-6-sulfonic acid (ABTS) assays have been frequently used to evaluate the antioxidant potential of these species.

Onyancha et al. [27] assayed rhizome methanolic extract of *H. abyssinica* using DPPH assay. This fraction showed higher activity of IC_50_ value of 26.7 µg/mL compared with the ascorbic acid (standard) which had an IC_50_ of 29.3 µg/mL. The radical scavenging potential was concentration-dependent. Moreover, the antioxidant potential of three extracts of *H. africana* (water, methanol, and acetone) were evaluated [25]. In both ferric-reducing antioxidant potential (FRAP) and DPPH assays, methanol extract showed high activity while acetone displayed the lowest. In nitric oxide (NO) scavenging, water extract depicted a higher activity than the control standard (gallic acid). Methanol and acetone extracts had good activities as reported in the ABTS assay. The radical scavenging and antioxidant reducing potential depicted by this species can be wholly or partially linked with the high content of phenols, flavonoids, and tannins that were earlier reported in these species. Additionally, the polarity of the solvents might have influenced the results since the compounds dissolved in them differently according to their polarity.

### 8.6. Antiproliferative Activity

Globally, cancer is a major health concern [79]. The search for an effective cancer cure is still on course since there is no specific chemotherapeutics for this dreadful disease. Stem cell treatment, radiotherapy, surgery, and chemotherapy are some of the commonly used cancer treatments [80]. They are linked with adverse effects such as excessive bleeding and non-specificity, as well as being ineffective and, more so, expensive [81]. This fact has influenced and propelled the medicinal research fraternity to look for alternatives to curb the high mortalities and morbidity associated with this disease. Herbs and other botanical products have been used to cure such diseases as they are considered safe and easy to administer with minimal chances of causing adverse reactions.

*H. abyssinica* rhizome was assayed using the 3-(4,5-dimethylthiazol-2-yl)-diphenyl tetrazolium bromide (MTT) assay on breast cancer (HCC) cells in vitro [65]. The methanol fraction depicted moderate anticancer activity of IC_50_ value 27.20 ± 1.1 µg/mL with high selectivity of SI = 3.68. Water extract showed low activity of IC_50_ value 499.3 ± 1.3 µg/mL. Water and ethanol extract of *H. abyssinica* root displayed low cytotoxicity against human KB cell lines with IC_50_ values > 50 µg/mL [65]. No antitumor activity was observed in the isolated compounds. Similar findings were reported in other studies [20,24,29]. Wintola et al. [82] performed an in vivo study and established that the safe dose of *H. africana* root extract was 5000 mg/kg body weight in both male and female laboratory rats when administered orally. This conclusion was drawn after 4 weeks of treating the animal models with the same dose with no reported cases of adverse reactions.

### 8.7. Antidiarrheal Activity

This is the frequent passage of semi-solid loose stool. It is characterized by dehydration and loss of minerals from the body. Some of its causes are bacterial infection, food/water contamination by parasites, medication side effects, and food poisoning [83]. Some antidiarrhea drugs bind to the receptors of pathogens with high affinity reducing their virulence. Other drugs coat the gastrointestinal (GIT) wall, minimizing the flow of fluids and ions in the gut and intestines, allowing absorption to take place [84]. Plants could provide a long-term solution for different ailments such as diarrhea, especially *H. abyssinica*.

As mentioned earlier, the *Hydnora* species have good antibacterial activities, implying their capability to minimize diarrhea which is implicated as an opportunistic disease of bacterial infection such as *Escherichia coli* [69]. An in vivo study conducted using albino rats demonstrated that indeed *H. abyssinica* root extract has antidiarrheal activity [85]. Firstly, diarrhea was induced in the animals by feeding them with castor oil. Later, they were treated using different sample doses ranging from 200 to 400 mg/kg. Those that received 400 mg/kg dosage showed the highest antidiarrhea inhibition of 74% and 60% after 4 and 6 h, respectively. The activity was concentration-dependent, but not time-dependent. This activity was linked to the inhibition of prostaglandins and other diarrhea inducers implicated in cytoprotective ability as previously reported [85]. Moreover, since not all pharmacological activities of this genus have been fully explored as well as the clinical evaluations of the crude preparations and isolated compounds, this creates an avenue for more research.

## 9. Conclusions and Future Perspectives

This review pointed out the benefits of *Hydnora* species in the traditional medicinal applications among different communities in Africa and Asia especially in treating various diseases. Previous studies have reported different classes of bioactive compounds isolated from only *H. abyssinica* which contain several pharmacological properties. No such studies have been conducted on the other species of this genus which remain hardly studied.

Regardless of these species commonly used as a remedy for gastrointestinal, diarrhea, and throat inflammations, the knowledge on their morphological features in the past was unknown, for instance, rhizomes resembling *H. abyssinica* (“*mavumbule*”) were traded in Mozambique markets. However, the traders had inadequate information on the appearance of the flowers and where the plant grew [86]. Therefore, the selected parts of the *Hydnora* species used for curing various ailments should be scrutinized to avoid further confusion and wrong administration.

A previous study indicated that *H. abyssinica* was used together with other medicinal plants to increase its efficacy in curing several ailments. For example, when mixed with *Justicia exigua* S. Moore leaves, it is used to cure paralysis. When combined with *Microglossa angolensis* Oliv. and Hiern (= *Conyza pyrrhopappa* Sch. Bip. ex A. Rich.) roots, it is used to treat diabetes. Further, when combined with *Passiflora edulis* Sims leaves, it is used to treat hemorrhoids [56]. However, the clinical application of these combinations has not been confirmed. Additionally, the pharmacological action mechanisms underlying the synergistic effects of these species and their medicinal material applications are yet to be explored and also their therapeutic properties remain unknown. Thus, it is highly recommended to test the clinical applications of *Hydnora* when combined with other medicinal plants in future research. The information available on *Hydnora* species indicates that they are less toxic. Therefore, dosages administered should be carefully determined to avoid adverse effects. Furthermore, most of the studies conducted on *H. abyssinica* and *H. africana* focused majorly on their rhizomes. Consequently, future studies should be conducted to evaluate and analyze the biological activities of their leaves, seeds, and stems.

Moreover, no specific comprehensive studies on the cultivation, processing, quality, and management of *Hydnora* species. Only *H. africana* could be cultivated once outside its native area on *Euphorbia* caput-medusae in California. Additionally, trials to cultivate *H. africana* in the University of Bristol Botanic Garden (UK) since 2008 using seeds planted on *Euphorbia tirucalli* L. have been unsuccessful [12]. Hence, more trials to cultivate these plants will enhance understanding of the life history of this important genus and conservation purposes. This will be helpful to populations, which are endemic to endangered environments, for example, *H. arabica* in the Arabian Peninsula and *H. esculenta* in Madagascar.

*Hydnora* being the oldest in the parasitic lineage provides a gap for further research in studying the evolutionary origin of their parasitic nature and more molecular research work is encouraged. Recently, molecular studies on this genus have indicated a spotlight using plastome to understand evolution in parasitic plants. Moreover, more research work is necessary to understand the life history and biology as well as the diversity of this genus.

In conclusion, this review paper summarized the past and current research studies on the ethnobotanical uses, phytochemistry, pharmacological activities, and distribution of the genus *Hydnora.* However, further research studies are encouraged to explore the efficacy of the identified bioactive compounds, and understanding the toxicity of these medicinal plants as well as confirming their safety for clinical use. Additionally, as an essential medicinal holo-parasitic plant in both Africa and the Arabian Peninsula, the ethnobotanical identities of the six remaining species of *Hydnora* and their pharmacology, toxicology, and phytochemistry are underexplored, thus requiring more research attention.

## Figures and Tables

**Table 1:** Ecological conditions and host.

Species	Ecological Conditions and Host	References
*H. abyssinica*	Found in dry woodland, wooded grassland, or bushland Host plants include Acacia *karoo* Hayne, *A. nilotica* (L.) Delile, *A luederitz* Engl., *A. tortilis* (Forssk.) Hayne, *A*. *seyal* Delile, *A*. gerradii Benth., *A. karoo* Hayne, *A. xanthophloea* Benth., *A. grandicornuta* Geratner, *A. tortilis* subsp. *heteracantha* (Burch.) Brenan and *A. nigrescens* Oliv.	[6,40]
*H. africana*	Found in the semi-arid arid and dry regions Host plants are *Euphorbia mauritanica* L., *E. tirucalli* L., *E. caputmedusae* L., *E. indecora* N.E.Br. (=*Euphorbia decussata* E.Mey. ex Boiss), *E. gregaria* Marloth, *E. gummifera* Boiss, *E. karrounsis* (Bois.) N.E Br, *E. lignosa* Marloth, *E. mauritanica* L., and *Albizzia lebbek* (L.) Benth.	[8,12]
*H. arabica*	It grows above the ground surface only when flowering Host plants; *Acacia tortilis* (Forssk.) Hayne and *Pithecellobium dulce* (Roxb.) Benth.	[1]
*H. sinandevu*	Found in scattered tree grassland, *Commiphora* bushland, thicket, or forest margin between mangrove and forest Host plants; *Commiphora campestris* Engl. and *C. africana* (A.Rich.) Endl. roots, as well as on *Pterocarps* or *Ostryodris*	[10,11]
*H. visseri*	Found in succulent karoo and Nama-Karoo vegetation in Namibia and the Northern Cape region of South Africa Host plants; *Euphorbia gregaria* Marloth, and *Euphorbia gummifera* Boiss.	[5]
*H. esculenta*	Found in semi-arid and dry areas in East Madagascar Mostly parasitizing Fabaceae species, *Albizzia tulearensis* R.Vig., and *Pithecellobium dulce* (Roxb.) Benth.	[7]
*H. triceps*	It is found in South Africa and Namibia Host plants are *Euphorbia dregeana* E. Mey. ex Boiss., and *Zygophyllum orbiculatum* Welw. ex Oliv.	[6,9]
*H. longicollis*	Found in Angola Host plants include *Zygophyllum orbiculatum* Welw. ex Oliv., *Euphorbia damarana* L.C.Leach, and other *Euphorbia* species	[5]

**Table 2:** The uses of *Hydnora* species.

Species	Use	References
*H. africana*	Food, tanning leather, fishing nets preservation, treat dysentery, chronic diarrhea, stomach crumps, stop bleeding, kidney and bladder, treat acne, inflamed throat	[14,51]
*H. abyssinica*	Food, treat dysentery, diarrhea, cholera, swelling of tonsillitis, charcoal, tanning leather, paralysis, diabetes, hiccups, fever, insomnia, hypertension, measles, hemorrhoids, throat inflammation, styptic gastric ulcer, and cancer	[6,24,52,53]
*H. visseri*	Food, treatment of diarrhea, hypertension, and diabetes	[5]
*H. esculenta*	Source of food	[4,7]
*H. arabica*	Source of food (fruits)	[1]
*H. triceps*	Food for wild animals	[9]
*H. sinandevu*	Treatment of throat infections	[51]
*H. longicollis*	Food for wild animals	[5]

**Table 3:** Chemical compounds characterized in *H. abyssinica.*

Chemical Classes	Compounds	Plant/Part(s)	Characterization Method	References
Phenylpropanoids	Katsumadin	*H. abyssinica* rhizomes	NMR	[74]
Flavonoids	Catechin	*H. abyssinica* (whole plant and roots)	NMR	[20,73,74]
	Cirsiliol			
	Trans 3′ 5-dihydroxy- 4′ 7-dimethoxydihydroflavonol			
Esters	2-hydroxyhexadecyl ester	*H.* *abyssinica*	NMR/ GC-MS	[20,26]
	Benzoic acid, 3, 4, dihydroxy-, ethyl ester			
Fatty acids	Myristic acid	*H. abyssinica* (whole plant and roots)		
	Oleic acid	*H. abyssinica* roots		[74]
	Palmitic acid	*H. abyssinica* roots	GC-MS	
Phenolic acids/derivative	Tyrosol	*H. abyssinica* (plant and roots)	NMR	[20,72,74]
	Protocatechuic acid			
Aldehydes	Vanillin			
Monoterpenes	Rhodioloside (glycoside)	*H. abyssinica* rhizomes	NMR	[74]
	Sabinene	*H. abyssinica* flowers	GC-MS	[24]
	D-limonene			
	γ-Terpinene			
	α-Terpinene			
Sterols	stigmasterol	*H. abyssinica* roots		[74]
Organic acids	Acetic acid	*H. abyssinica* flowers	GC-MS	[24]

**Table 4:** Antibacterial and antifungal activities of *H. abyssinica* and *H. africana.*

Plant/Part Investigated	Assay Method	Results	References
*H. abyssinica* flowers	Agar diffusion	All extracts showed low activity on yeast, Gram-positive, and Gram-negative bacteria	[24]
*H. abyssinica* rhizome	Agar disc diffusion/well diffusion	In both assays, the methanol and methanol-dichloromethane extracts exhibited moderate to high activities against bacteria tests. Their highest activity was against *Candida albicans*. Water extract showed relatively low activity. Generally, the inhibition activities were dose-dependent	[29]
*H. abyssinica* rhizome	Cup-plate agar diffusion	This sample showed promising antibacterial activity against the four bacteria and fungi strains assayed; however, it showed no inhibition against *Sesbania leptocarpa*	[67]
*H. abyssinica* rhizome	Disk diffusion	The crude extract had the highest activity of >20 mm minimum inhibition diameter	[63]
*H. abyssinica* root	Disk diffusion	The methanol extract exhibited low antibacterial activity. No activity was observed at 6.5 and 12.5 mg/mL sample concentrations on *Bacillus subtillis*	[21]
*H. abyssinica* root	Cup-plate agar diffusion	Methanol, chloroform, and petroleum ether exhibited partial to high antibacterial activity on all strains tested except on *Pseudomonas aeruginosa* where no activity was observed. High inhibition zones were observed in methanol extract. The activities were dose-dependent	[66]
*H. abyssinica* root	Cup-plate agar diffusion	The extracts’ activity increased with an increase in concentration. The water extract exhibited higher inhibition against all fungi and bacteria strains with >16 and >6 mm, respectively. A weak activity was observed in chloroform	[64]
*H. abyssinica* root	Broth microdilution	The water extract exhibited high activity against *Enterococcus faecalis, Bacillus cereus,* and *Bacillus subtilis* with MIC values of 16 and 64 µg/mL, respectively. Low activity was observed in all other antibacterial tests	[20]
*H. africana*	Agar well diffusion	The MIC_50_ of methanol, acetone, ethanol, and ethyl acetate extracts ranged from 0.078–2.5 mg/mL. Methanol extract had no inhibition against *H. pylori*	[27]
*H. africana*	Dilution microplate	The sample showed moderate activity against all bacteria strains. The activity was both time and concentration-dependent	[69]
*H. africana*	Agar well diffusion	Acetone and ethanol extract exhibited moderate to high MIC as compared to ciprofloxacin (drug). The aqueous extract showed no activity. The ethanol extract was highly active on both *Escherichia coli* and *Pseudomonas aureginosa.* Acetone had high activity against *Shigella sonnei*	[63]
